# Early predictors of language outcomes in Down syndrome: A mini-review

**DOI:** 10.3389/fpsyg.2022.934490

**Published:** 2022-09-14

**Authors:** Marisa G. Filipe, Sara Cruz, Andreia S. Veloso, Sónia Frota

**Affiliations:** ^1^Center of Linguistics, School of Arts and Humanities, University of Lisbon, Lisbon, Portugal; ^2^Universidade Lusófona do Porto, Porto, Portugal; ^3^Faculty of Psychology and Education Sciences, Center for Psychology at University of Porto, University of Porto, Porto, Portugal

**Keywords:** Down syndrome, systematic review, early predictors, language, impairments

## Abstract

As children with Down syndrome (DS) typically manifest significant delays in language development, the research has pointed out the predictors of later language skills for this clinical population. The purpose of this study was to systematically explore the evidence for early predictors of language outcomes in infants and toddlers with DS from studies published between 2012 and 2022. After the search, nine studies met the inclusion criteria. The results indicated that maternal educational level, adaptive level of functioning, cognitive function, attention skills, communicative intent of the child, early vocalizations, gestures, baby signs, parents’ translation of their children’s gestures into words, and vocabulary level are significant predictors of language outcomes in children with DS. These findings provide a timely and warranted summary of published work that contributes to current understanding of the development of language and communication in DS. They are therefore useful to researchers, clinicians, and families.

## Introduction

As language is crucial for learning and academic achievement (Johnson et al., 2010; Conti-Ramsden et al., 2018; Eadie et al., 2021), the development of language skills is essential to meet the increasing demands of modern societies (Duncan et al., 2007). Indeed, research has shown that children with low language abilities are at high risk of difficulties with literacy, academic achievement, and social-emotional and behavioral adjustment (Voci et al., 2006; Zubrick et al., 2007; Tromblin, 2008; Durkin and Conti-Ramsden, 2010; Johnson et al., 2010; Conti-Ramsden et al., 2018). Thus, research on language development is particularly useful.

Previous studies have identified typical trajectories for language development. For example, at the age of 10–12 months, children can discriminate phonemes in their native language (for a review, refer to Kuhl, 2010), begin to understand and utter words, and produce representational and deictic gestures (Fenson et al., 1994; Caselli et al., 2012). At 18 months, typically developing (TD) children reach a lexical repertoire of approximately 50 words and use gesture–word combinations frequently (Fenson et al., 1994; Capirci and Volterra, 2008). Between 20 and 24 months, they increase expressive vocabulary and start to combine words (Fenson et al., 1994; Capirci and Volterra, 2008). Children at the age of 3 years have been found to produce a more complex lexicon, as well as utterances that are grammatically more accurate and richer (for a review, see Guasti, 2017).

Identification of these typical language trajectories is important as many children can experience language delays (Reilly et al., 2007; Zubrick et al., 2007), as a result of biological, cognitive, and environmental factors (Kuhl, 2010; Perani et al., 2011; Riva et al., 2017). In fact, several children diagnosed with neurodevelopmental disorders have language specificities and may later be diagnosed with language impairments. For example, children with Down syndrome (DS; which results from a partial or complete duplication of chromosome 21; Epstein, 1986) display a complex neurocognitive profile including particular patterns of language skills that are characterized by relative strengths and weaknesses. On the one hand, receptive vocabulary (Laws et al., 2015) and the use of gestures (Iverson et al., 2003) appear as relative strengths in the language profile of children with DS. But on the other hand, children with DS frequently display severe language difficulties (Abbeduto et al., 2007a) and are less likely to accompany prelinguistic communicative gestures with vocalizations when compared to TD peers matched by their sensorimotor development (Greenwald and Leonard, 1979). Children with this clinical condition also tend to produce their first words at approximately 21 months (Stoel-Gammon, 2001) in line with their cognitive abilities (Miller, 1999), and expressive language abilities can be delayed when compared to receptive language and non-verbal skills (Chapman and Hesketh, 2000; Abbeduto et al., 2007b). Furthermore, in DS, the development of word segmentation competencies is seriously compromised (Mason-Apps et al., 2018), infants with DS do not use prosody as a facilitator for word segmentation unlike TD infants (Frota et al., 2020), reduced speech intelligibility is common (Kumin, 1994; Kent and Vorperian, 2013), and more substantial delays in expressive syntax than in expressive vocabulary have been reported (Kover et al., 2012). Longitudinal studies have also suggested that vocabulary development in DS is slower compared to the language development of TD peers, which, in turn, seems to be related to general cognitive abilities (Cuskelly et al., 2016; Kaat-van den Os et al., 2017).

To understand how different variables impact development and predict which children are most likely to have language impairments, researchers are identifying early predictors of language trajectories in different subgroups of community cohorts (McKean et al., 2017). In fact, several environmental and child-related factors associated with language delays or impairments have been found, such as male gender, prematurity, low birth weight, perinatal disorders, low income, and low parental education (Nelson et al., 2006; Sansavini et al., 2010; Snowling et al., 2016; Bishop et al., 2017). Other variables may also predict language outcomes in typical and atypical development. For instance, non-verbal requesting is a longitudinal predictor of expressive language development (e.g., Mundy et al., 1995) and prelinguistic communication reveals children’s readiness to acquire language while eliciting language-facilitating responses from parents (Yoder and Warren, 1993; Yoder et al., 1998). Auditory and visual processing in early speech perception has also been shown to be crucial to language outcomes (Friederici, 2006; Kuhl et al., 2008; Ellis et al., 2015), affecting speech segmentation, word learning, and phrase-level processing.

Regarding DS, it is often suggested that the same environmental and child-related predictors found for TD children apply to children with this condition (e.g., Deckers et al., 2019). Indeed, previous studies found that gestures predict language development in children with typical development and DS (Capone and McGregor, 2004; Rowe et al., 2008; Zampini and D’Odorico, 2009). In addition, (i) the use of gestures at 24 and 36 months of age has been shown to predict future vocabulary growth (Zampini and D’Odorico, 2009), (ii) early prosodic development predicted lexical development in similar ways for infants and toddlers with typical development, at-risk for language impairments, or with DS (Sousa et al., 2022), (iii) babbling correlated with later language development (Locatelli et al., 2021) in line with previous studies on TD children (e.g., Lang et al., 2019), and (iv) the relationship between motor and language development was found to become stronger as the age of children increases (Yamauchi et al., 2019), a pattern that is also consistent with findings for TD children (e.g., Alcock and Krawczyk, 2010).

However, research has also suggested that different variables might predict language development in children with DS. Mason-Apps et al. (2018) showed that (i) non-verbal mental skills were the significant longitudinal predictors of language for infants with DS but not for TD infants, (ii) speech segmentation abilities only predicted language outcomes in the TD group, and (iii) while initiating joint attention was critical for TD participants, response to joint attention was more predictive of language scores in infants with DS than in TD participants. Indeed, research has shown important differences in early visual attention abilities and audiovisual speech processing in infants with DS compared to typically developing infants (D’Souza et al., 2016; Pejovic et al., 2021).

As several predictors of language outcomes have been reported in children with DS, the aim of this study was to systematically review the articles that focus on early precursors of language in infants with this genetic condition. We will focus on early predictors that appear before 30 months of age, given the potential of early screening to identify children at risk of developing language difficulties in the first 2 years of life (Määttä et al., 2012). Understanding these early predictors of language variability is important to determine the factors that explain why some children with DS acquire language before others (Sameroff and Chandler, 1975). This could also contribute to the development of an early intervention that facilitates language learning in young children, which is strongly recommended due to the link between language skills and later development (e.g., Luyster et al., 2007).

## Methods

This study adopted the method of a systematic review, as required by the Cochrane Collaboration and the PRISMA framework (Moher et al., 2009). In March 2022, using EBSCOhost, the following databases were searched: Academic Search Complete, APA PsyArticles, ERIC, MEDLINE, ScienceDirect, and Psychology and Behavioral Sciences Collection. The keywords *language* AND *longitudinal* OR *prospective* AND *down syndrome* OR *trisomy 21* OR *down’s syndrome* were used to conduct the search. The following filters were applied: (i) publication date from 2012 to 2022, (ii) academic journals, and (iii) peer-reviewed. All titles/abstracts identified in the electronic databases were independently screened for eligibility by two authors (MF and SC), according to the following inclusion criteria:

• The study was published in a peer-reviewed journal from 2012 to 2022.
• Participants were followed for a period of 3 months or more in a prospective cohort study.
• The study design was experimental or observational.
• The report presented at least one early (collected before the first 30 months of age) and a later language measure/outcome.
• The subsequent result(s) should include at least one quantitative measure to compare the findings across the studies.
• The report was written in English.

**FIGURE 1:**
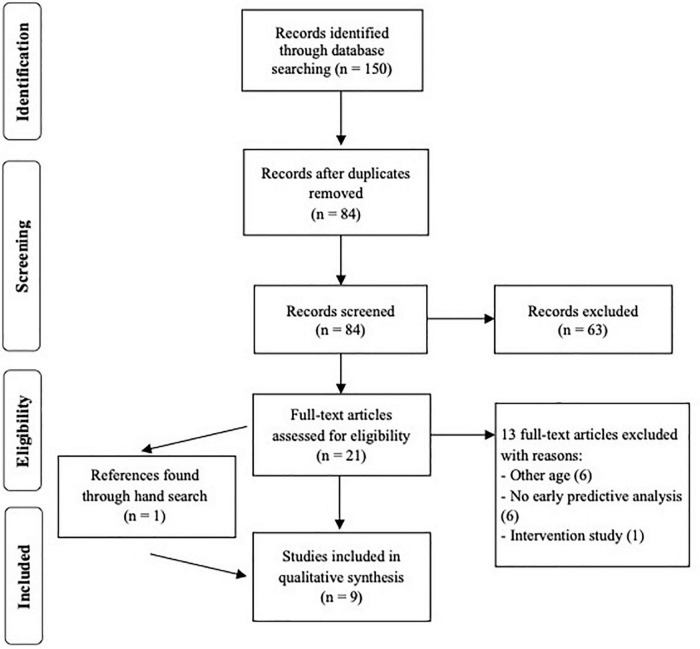
Preferred reporting items in systematic reviews and meta-analyses (PRISMA) flow diagram of the study selection procedure.

The search identified 150 articles. After the removal of duplicates, if the title and abstract suggested that the study may be appropriate for inclusion, the full-text article was evaluated according to the previously established inclusion criteria. A total of 21 articles were selected for full-text review. Hand searches, which included checking the reference lists of the included journal articles, identified another paper which was also read in full. A total of nine studies were included in the mini-review. Percentage agreement on the selection of included studies was 95.51%. Percentage agreement after consensus building was 100%. The selection of studies is depicted in Figure 1 in a PRISMA flow diagram (Moher et al., 2009). The list of excluded studies along with reasons for exclusion are presented in Supplementary material.

From each eligible study, the following data were extracted: first author name, publication date, study location, primary language, number of participants, age at intake, time to follow-up, language predictors, language predictor measures, language outcomes, language outcome measures, main findings, and effect sizes.

## Results

A summary of each study characteristics is presented in Table 1. The sample sizes of children with DS ranged from 5 to 26 participants. Almost half of the studies were conducted in the United States, and the remaining studies were carried out in different countries including the Netherlands, Sweden, Italy, and the United Kingdom. Age at intake varied between 10 and 84 months, with follow-ups conducted 6–53 months later.

**TABLE 1:** Characteristics of the included studies.

Authors (year) location, language	Participants (n)	Age at intake	Age at follow-up	Measures and predictors of language development	Measures and language outcomes	Main findings
Deckers et al. (2019) Netherlands, Dutch	DS: *N* = 20	2.0–7.0 years	+ 1.6 years or 18 months	**Measure:** Vineland Screener **Predictor:** Adaptive level of functioning **Measure:** Subscale Working Memory from the Behavior Rating Inventory of Executive functions—Preschool version **Predictor:** Working memory **Measure:** Child Behavior Checklist 1.5–5 **Predictor:** Behavioral and emotional problems, attention distractibility and temperament **Measure:** The Bridge: Emergent literacy skills **Predictor:** Book reading experiences and phonological/phonemic awareness **Measure:** Social Networks Questionnaire **Predictor:** Number of communication partners **Measure:** Sociodemographic Questionnaire **Predictor:** Socioeconomic status, chronological age of the child, siblingship size, educational level, and involvement of the child **Measure:** Receptive One-word Picture Vocabulary Test **Predictor:** Receptive vocabulary **Measure:** Auditory Discrimination Task **Predictor:** Auditory discrimination **Measure:** Auditory Working Memory Test **Predictor:** Auditory working memory **Measure:** Communicative Intentive Onderzoek **Predictor:** Communicative intent, joint attention and parental support and responsiveness	**Measure:** MacArthur Communicative Development Inventories **Outcome:** Expressive vocabulary **Measure:** Receptive One-word picture Vocabulary Test **Outcome:** Receptive vocabulary	• Expressive vocabulary development was best predicted by the adaptive level of functioning (*R*^2^ = 0.80; *p* = 0.01), receptive vocabulary (*R*^2^ = 0.73; *p* = 0.001), maternal educational level (*R*^2^ = 0.42; *p* = 0.01), level of communicative intent of the child (*R*^2^ = 0.53; *p* = 0.01), attention skills (*R*^2^ = 0.63; *p* < 0.05), and phonological/phonemic awareness (*R*^2^ = 0.69; *p* = 0.01). • Receptive vocabulary development was best predicted by the adaptive level of functioning (*R*^2^ = 0.88; *p* = 0.001) and early receptive vocabulary skills (*R*^2^ = 0.84; *p* = 0.001).
Dimitrova et al. (2016) USA, English	TD: *n* = 23 Autism: *n* = 23 DS: *n* = 23	TD: 18–30 months Autism: 31–43 months DS: 30–45 months	± 12 months	**Measure:** Communication Play Protocol **Predictor:** Parents’ translations of child gesture	**Measure:** Communication Play Protocol **Outcome:** Expressive vocabulary development	• Parents translate a high percentage of their children’s gestures into words, and this input was beneficial for children in each group as they acquire more words for the translated gestures than the not translated ones. Translation: *F*(1, 63) = 5.97, *p* = 0.02, ν^2^*_*p*_* = 0.09 . Group: *F*(2, 63) = 8.01, *p* = 0.001, ν^2^*_*p*_* = 0.20 . Group × Translation: *F*(2, 63) = 0.05, *p* = 0.95 • This benefit on child vocabulary development was particularly evident for children who show evidence of vocabulary growth over time. . Translation: *F*(1, 45) = 6.63, *p* = 0.013, ν^2^*_*p*_* = 0.13 . Group: *F*(2, 45) = 6.54, *p* = 0.003, ν^2^*_*p*_* = 0.23 . Group × Translation: *F*(2, 45) = 0.30, *p* = 0.743
						• The use of these spoken labels had the same facilitative effect on vocabulary development for children with TD and DS.
Kaat-van den Os et al. (2017) Netherlands, Dutch	DS: *N* = 26	18–24 months	Monthly assessments over an 18-month period	**Measure:** Cognition Scale of the Bayley Scales of Infant and Toddler Development, Third Edition **Predictor:** General cognitive function	**Measure:** Lexi Questionnaire **Outcome:** Expressive vocabulary growth and modality (gesture and/or verbal production)	• Three patterns of vocabulary growth were identified: children with a marginal vocabulary growth, children with an increase in vocabulary without a growth spurt, and children who showed a vocabulary growth spurt. • All groups significantly differed in the rate of vocabulary growth. . Growth spurt (GS): *M* = 56.2, *SD* = 52.9 . Without growth spurt (WGS): *M* = 3.9, *SD* = 2.9 . Marginal growth pattern (MGP): *M* = 1.1, SD = 0.6 - GS vs. WGS: *p* < 0.05 - WGS vs. MGP: *p* < 0.01 • The general cognitive function of the children with a marginal growth pattern was significantly lower than that of the children in the groups with a substantial increase in vocabulary or vocabulary spurt. . GS: *M*_age_ = 19 . WGS: *M*_age_ = 18.5 . MGP: *M*_age_ = 15.9 - GS vs. MGP: *p* < 0.05 - WGS vs. MGP: *p* < 0.05 • The general cognitive function of the groups with or without a growth spurt did not differ significantly. • Correlation showed that the rate of vocabulary growth was significantly correlated with the general cognitive function (*r* = 0.44, *p* < 0.05).
Mason-Apps et al. (2018) United Kingdom, English	DS: *n* = 14 TD: *n* = 35	10–19 months	Measures collected at two time points, approximately 6 and 12 months apart from intake	**Measure:** Mullens Scales of Early Learning **Predictor:** Non-verbal mental ability **Measures:** Strong-Weak Task (to assess infants’ ability to segment bisyllabic words with a strong-weak stress pattern) and Weak-Strong Task (to assess the ability to segment bisyllabic words with a weak-strong stress pattern) **Predictor:** Speech segmentation skills **Measure:** Early Social Communication Scales **Predictor:** Social communication skills (initiating and responding to joint attention; initiating behavioral requests)	**Measure:** Preschool Language Scales-4 **Outcome:** Auditory comprehension and expressive communication **Measure:** Reading Communicative Development Inventory **Outcome:** Receptive and expressive vocabulary	• In the TD group, speech segmentation and initiating joint attention were the strongest predictors of later language. . Speech segmentation (SS; T1) × expressive communication (EC; T2): *r* = 0.701, *p*≤ 0.001 . SS (T1) × expressive vocabulary (EV; T2)*: r* = 0.553, *p*≤ 0.01 . Initiating joint attention (IJA; T1) × expressive communication (EC; T2): *r* = 0.490, *p*≤ 0.05 . IJA (T1) × EV (T2): *r* = 0.402, *p*≤ 0.05 - Regression analysis (EC, SS, IJA, age): *F*(4, 15) = 18.17, *p* < 0.001, Adj*R*^2^ = 0.783 - Regression analysis (EV, SS, IJA, age): *F*(3, 18) = 5.68, *p* = 0.006, Adj*R*^2^ = 0.401
						. SS (T1) × auditory comprehension (AC; T3): *r* = 0.498, *p*≤ 0.05 . SS (T1) × EC (T3)*: r* = 0.685, *p*≤ 0.001 . SS (T1) × receptive vocabulary (RV; T3)*: r* = 0.565, *p*≤ 0.05 . SS (T1) × EV (T3)*: r* = 0.827, *p*≤ 0.001 . IJA (T1) × EV (T3): *r* = 0.413, *p*≤ 0.05 - Regression analysis (EC, SS, age): *F*(3, 17) = 7.04, *p* = 0.003, Adj*R*^2^ = 0.475 • In the DS group, non-verbal mental ability and responding to joint attention were the strongest predictors of later language. . Non-verbal mental ability (NVMA; T1) × AC (T2): *r* = 0.862, *p*≤ 0.001 . NVMA (T1) × Receptive vocabulary (RV; T2): *r* = 0.855, *p*≤ 0.01 . Non-verbal mental ability (NVMA; T1) × RV (T3): *r* = 0.871, *p*≤ 0.001 . Responding to JA (RJA; T1) × AC (T3): *r* = 0.614, *p*≤ 0.01 . RJA (T1) × EC (T3): *r* = 0.812, *p*≤ 0.001 . RJA (T1) × RV (T3): *r* = 0.629, *p*≤ 0.05 . RJA (T1) × EV (T3): *r* = 0.656, *p*≤ 0.05 - Regression analysis (NVMA, RJA, RV, age): *F*(4, 7) = 12.662, *p* = 0.003, Adj*R*^2^ = 0.809 - Regression analysis (EC, RJA, age): *F*(1, 10) = 11.906, *p* = 0.002, Adj*R*^2^ = 0.645 • Non-verbal mental skills were a significant longitudinal predictor of language for infants with DS but not for TD infants, speech segmentation abilities only predicted language outcomes in the TD group, and while initiating joint attention was critical for TD participants, response to joint attention was more predictive of language scores in infants with DS than in TD participants.
Nyman et al. (2021) Sweden, Swedish	DS: *n* = 5 Cerebral palsy (CP): *n* = 4 Chromosomal deletion syndromes: *n* = 2	12–22 months	4:11–5:4 years	**Measure:** Audio-video recordings of parent–child interaction, using a standardized procedure and set of toys. A babbling observation was performed, and the occurrence of different babbling variables was noted using an observation form containing a list of all 18 Swedish consonant sounds. **Predictor:** Consonant use	**Measure:** Test for Reception of Grammar-2 or Reynell Developmental Language Scales-III **Outcome:** Receptive language **Measure:** The five longest utterances for each child were identified based on all spontaneous communication. Mean maximum utterance length was calculated by taking the five longest utterances, adding up the number of words and dividing it by five **Outcome:** Expressive language **Measure:** Expressive Vocabulary and Sentence Recall from the Clinical Evaluation of Language Fundamentals-4 **Outcome:** Expressive language **Measure:** Swedish Communicative Development Inventory III or Swedish Communicative Development—words and gestures **Outcome:** Number of words the child understands and produces	• Children with DS performed lower than participants with other types of neurological disabilities on two consonant production measures of the Swedish Articulation and Nasality Test. . Percentage of consonants correct (PCC): DS vs. CP: *U* = 0, *p* = 0.016 . Number of established consonants: DS vs. CP: *U* = 1.5, *p* = 0.032 • However, participants with DS who used a high number of different true consonants at the first assessment also had higher consonant production measured at the follow-up. . Correlation (*n* true consonants at T1 × PCC at T2): *r*_s_ = 0.553, *p* = 0.077 . Correlation (*n* true consonants at T1 × PCC at T2 – DS subgroup analysis): *r*_s_ = 0.894, *p* = 0.041
					**Measure:** Swedish Articulation and Nasality Test **Outcome:** Consonant Production **Measure:** Presence of motor speech disorder was assessed based on the audio and video recorded articulation test **Outcome:** Presence of motor speech disorder **Measure:** Intelligibility in Context Scale **Outcome:** Functional intelligibility	
Özçalışkan et al. (2017) USA, English	DS: *n* = 23 TD: *n* = 23 Autism (ASD): *n* = 23	DS: 30 months TD: 18 months ASD: 30 months	5 times over a year	**Measure:** Communication Play Protocol **Predictor:** Referents expressed uniquely in gesture	**Measure:** Communication Play Protocol **Outcome:** Referents later expressed in speech	• A significant positive correlation was found between the age at which a child expressed referents uniquely in gesture and the mean age they were expressed later in speech across the three groups and within each group. . Correlation (across all groups): *r* = 0.93, *p* < 0.001 . Correlation (ASD): *r* = 0.87, *p* < 0.001 . Correlation (DS): *r* = 0.81, *p* < 0.001 • Most of the referents conveyed uniquely in gesture entered children’s spoken vocabularies as words for both TD children and children with autism within a year. This pattern was less pronounced for children with DS, who differed significantly from both groups. . Modality shift from gesture to speech: *F*(1, 63) = 4.46, *p* = 0.04, η^2^*_*p*_* = 0.07 . Interaction between group and modality shift: *F*(2, 63) = 6.45, *p* = 0.003, η^2^*_*p*_* = 0.17 • The time interval from when a referent was observed in gesture and its observation in speech was longer for DS compared to TD. . Timing of the modality shift from gesture to speech: - modality: *F*(1, 48) = 427.92, *p* < 0.001, η^2^*_*p*_* = 0.90 - group: *F*(2, 48) = 92.36, *p* < 0.001 η^2^*_*p*_* = 0.79 - interaction between group and modality: *F*(2, 48) = 9.52, *p* < 0.001, η^2^*_*p*_* = 0.28
Özçaliskan et al. (2016) USA, English	DS: *n* = 23 TD: *n* = 23	DS: 2.6 TD: 1.6	+ 12 months	**Measure:** Communication Play Protocol **Predictor:** Gestures and signs (deictic, conventional, iconic)	**Measure:** Previously transcribed transcripts **Outcome:** Spoken vocabulary **Measure:** Expressive Vocabulary Test **Outcome:** Vocabulary size	• For children with DS, the production of baby signs predicted expressive vocabulary size 1 year later (*Spearman’s rho* = 0.60, *p* = 0.005). Neither deictic nor conventional gestures produced by children with DS had a significant relation to later spoken vocabulary. • Deictic gestures reliably predicted expressive vocabulary size for TD children (*Spearman’s rho* = 0.64, *p* = 0.002), while baby signs were positively related to later vocabulary of children with DS.
Zampini and D’Odorico (2013) Italy, Italian	DS: *N* = 18	Ten 2-year-old children Eight 3-year-old children	2-year-old children were followed for a 2-year period 3-year-old children were followed for a 1-year period	**Measure:** MacArthur–Bates Communicative Development Inventories (production checklist) **Predictor:** Vocabulary size **Measure:** Brunet–Lézine Scale of Psychomotor Development **Predictor:** Developmental level	**Measure:** MacArthur–Bates Communicative Development Inventories (production checklist) **Outcome:** Lexical outcomes	• Only at 36 and 42 months could vocabulary size explain individual differences on subsequent lexical development at 48 months, and only at 42 and 48 months could developmental age explain the variability in children’s lexical outcomes. . Lexical outcomes at 48 months and first stages of vocabulary acquisition: - 36 months × low outcome group × medium outcome group × high outcome group: *K* = 12.97, *p* = 0.002 - 42 months × low outcome group × medium outcome group × high outcome group: *K* = 15.05, *p* = 0.001 . Individual differences in children’s developmental ages and children’s lexical outcomes: - 42 months × low outcome group × medium outcome group × high outcome group: *K* = 7.67, *p* = 0.022 - 48 months outcome group × low outcome group × medium outcome group × high outcome group: *K* = 9.08, *p* = 0.011
Zampini et al. (2015) Italy, Italian	DS: *N* = 18	24 months	30 months	**Measure:** Semi-structured free-play sessions in interaction with their mothers **Predictor:** Joint attention	**Measure:** MacArthur-Bates Communicative Development Inventory **Outcome:** Vocabulary development (both receptive and expressive)	• The children’s behavior of proposing a joint attention focus to their communicative partners appeared to be a significant predictor of the children’s vocabulary comprehension skills as assessed 6 months later. . Total amount of time spent in joint attention and word comprehension: *r* = 0.577, *p* = 0.024 . Regressions: - Word comprehension at 24 months: *F*(1, 16) = 60.11, *p* < 0.001, *R*^2^ = 0.79, Adj*R*^2^ = 0.78 - Word comprehension at 24 months + joint attention propose + joint attention follow: *F*(2, 15) = 41.07, *p* < 0.001, *R*^2^ = 0.85, Adj*R*^2^ = 0.83

Several predictors of language outcomes were evaluated, namely, socioeconomic status, general cognitive function, developmental level, adaptative level of functioning, auditory working memory, attention skills, joint attention, behavioral and emotional problems, temperament, auditory discrimination, number of communication partners, level of communicative intent, book reading experiences, parents’ translations of child gestures, gestures, signs, initiation of behavioral requests, speech segmentation, consonant use, vocabulary, and phonological/phonemic awareness (cf. Table 1).

The following language outcomes were evaluated: consonant production, functional intelligibility, auditory comprehension, expressive communication, referents later expressed in speech, receptive and expressive vocabulary, vocabulary growth, and receptive and expressive language. Language measures varied between the studies. The MacArthur Communicative Development Inventories and the Communication Play Protocol were the most common language measures employed (cf. Table 1).

The results showed that most of the language outcomes were related to vocabulary. Regarding language predictors, adaptive level of functioning, vocabulary skills, maternal educational status, level of communicative intent of the child, attention skills, phonological/phonemic awareness (Deckers et al., 2019), parents’ translation of their children’s gestures into words (Dimitrova et al., 2016), baby signs (Özçaliskan et al., 2016), general cognitive function (Kaat-van den Os et al., 2017; including non-verbal mental ability: Mason-Apps et al., 2018), and joint attention (Zampini et al., 2015; responding to join attention: Mason-Apps et al., 2018) were the significant predictors of vocabulary skills. Non-verbal mental ability and responding to join attention were also the predictives of auditory comprehension (Mason-Apps et al., 2018). Furthermore, a significant positive correlation was found between the age at which a child expressed referents uniquely in gesture and the mean age they were expressed later in speech (Özçalışkan et al., 2017). Finally, a high number of different true consonants at early ages was associated with a higher consonant production measured at follow-up (Nyman et al., 2021).

## Discussion

This review contributed to a better understanding of early predictors (before 30 months of age) of language outcomes in children with DS. This enhances our theoretical understanding of language development by revealing the factors that underpin language acquisition. Identifying language predictors is critical to promote the early identification of individuals with language impairments. In general, the studies included in the review show that most children with DS make positive language gains that are evident in vocabulary measurements. Although it is difficult to draw strong conclusions based on the limited evidence available, it is becoming increasingly clear that early predictors of later language development may be present in the first 30 months of life. Based on the results of this review, the predictors of language outcomes in DS will be discussed in the following paragraphs.

Between 2 and 7 years of age, maternal educational level appears to be a predictor of later expressive vocabulary in DS (Deckers et al., 2019). Indeed, previous research had also suggested that mothers of TD children from a higher socioeconomic status used longer utterances and a more diverse vocabulary when talking to their toddlers, which was associated with greater vocabulary growth (Hoff, 2003).

Evidence for the adaptive level of functioning (i.e., the child’s level of participation in daily tasks involving conceptual, social, and practical skills) was also found as an early predictor of expressive and receptive vocabulary in children with DS, between 2 and 7 years of age (Deckers et al., 2019). This highlights that language development and the adaptative level of functioning might be interrelated. Indeed, previous studies with individuals with DS highlighted stronger skills in daily living activities and socialization compared with the relative weaknesses in motor and communication skills (e.g., Van Duijn et al., 2010). Probably, children with DS who are more likely to show social competence will elicit more reactions from communication partners, experience different social contexts, and learn more different words, while the use of language to communicate may in turn increase the ability to manage social situations. However, it is important to highlight that language development and the adaptative level of functioning are interrelated and growth in one skill might affect the functioning of the other.

We also found that, before 2 years of age, cognitive domains such as non-verbal mental ability (Mason-Apps et al., 2018), play skills, information processing, memory, habituation skills, and reasoning abilities (named by the authors as general cognitive function; Kaat-van den Os et al., 2017) predict vocabulary growth in DS. Furthermore, non-verbal mental ability was also found to predict auditory comprehension (Mason-Apps et al., 2018). Although research has shown that language outcomes in DS are not merely a result of a cognitive disability (e.g., Dodd and Thompson, 2001), the studies included in this review highlighted that several cognitive skills predicted language outcomes showing a clear link between cognitive skills and language learning. This is not surprising since domain-general abilities apply across different kinds of tasks (Federmeier et al., 2020).

A finding that is also evident in the present review is that attention skills found to predict language outcomes in TD children were also visible in children with DS. Namely, at 19 and 24 months of age (respectively, Zampini et al., 2015; Mason-Apps et al., 2018), joint attention predicted language outcomes, and between 2 and 7 years of age, attention skills predicted expressive vocabulary (Deckers et al., 2019). These results are in line with the previous studies for typically developing children. For instance, in TD 1-year-olds, the effect of maternal education and warm parenting on vocabulary growth was found to be mediated by attention skills and parent–child book reading when the children completed 3 years of age (Farrant and Zubrick, 2012). Furthermore, in TD individuals, higher attention demands negatively affect the aspects of spoken vocabulary (Hula et al., 2007). Thus, attention skills are important for language development in TD and in DS, probably because children with greater attention skills may be more likely to experience more opportunities for language learning.

We also found that, between 2 and 7 years of age, the level of communicative intent could be a predictor of later expressive vocabulary for children with DS (Deckers et al., 2019). Indeed, previous research has reported a result along similar lines for TD toddlers, showing that the level of communicative intent is a predictor of later language outcomes (Wetherby et al., 2002). Higher rates of communication could increase the opportunities for interaction and shape communication development (McCathren, 2000). For example, Yoder et al. (1994) showed that mothers provided more verbal modeling when children have a higher communicative intent.

Our findings also highlighted that consonant measures might be useful in evaluating toddlers with DS, namely, the number of true consonants assessed from 12 to 22 months of age might predict later consonant production (Nyman et al., 2021). A continuity between early vocalizations and language outcomes in atypical and typical development has been suggested in the literature. For instance, canonical babbling (which consists of consonant-vowel-syllables with a rapid transition between them) is commonly used in the study of early vocalizations in children at risk of language difficulties (Nyman et al., 2021). For TD, the early consistent use of consonants has also been associated with better expressive vocabulary (McGillion et al., 2017).

Children with DS are as likely as TD children to point to and request objects using gestures prior to using words, and our review highlighted that, at 30 months of age, the onset of referents expressed uniquely in gestures could predict the onset of similar spoken words (Özçaliskan et al., 2016). Also, at 1 year of age, parents’ translation of children’s gestures into words might predict later vocabulary development (Dimitrova et al., 2016). This is in line with what previous findings have suggested that parents gather information from the gestures their children produce and tailor their verbal responses to the communicative interests of the child (Goldin-Meadow et al., 2007; Olson and Masur, 2011). These parents’ translations of child gestures could help the child to map the word to the object of interest conveyed in gesture. Thus, children’s gestures probably provide cues to the parents about the child’s readiness to learn a particular word (Dimitrova et al., 2016).

Also related to gestures, an important finding is that baby signs (i.e., iconic or arbitrary signs intentionally taught by adults) at 2.5 years of age may be positively related to later vocabulary outcomes in children with DS (Özçaliskan et al., 2016). Baby signs are learned in the everyday context when a parent produces signs to refer to a particular object. The use of these repeated signed symbols might create a state symbol stand for objects (DeLoache, 2004) that could help children with DS to move from a repertoire of signed symbols to a repertoire of words. Thus, findings from this review seem especially significant considering current knowledge about the importance of early non-verbal communicative skills for the prediction of later language outcomes.

Finally, our results showed that a particularly important behavioral domain is the use of vocabulary skills as a key precursor to language development. Deckers et al. (2019) found that receptive vocabulary, between 2 and 7 years of age, was a predictor of later expressive and receptive vocabulary. A similar conclusion was reached by other studies. For instance, in children with DS, early receptive vocabulary skills tend to be a predictor of receptive and expressive vocabulary (Chapman et al., 2000; Chiat and Roy, 2008). However, Zampini and D’Odorico (2013) assessed children with DS from 2 years of age and showed that individual differences at 48 months could be explained by vocabulary size only at 36 and 42 months.

It seems that some predictors had the same facilitative effect for TD children and children with DS, such as the parents’ translation of gestures into words (Dimitrova et al., 2016). However, our review also emphasized that early predictors of language outcomes might be different for the two groups: (i) the time interval from when a referent was observed in gesture and its observation in speech was longer for DS compared to TD (Özçalışkan et al., 2017); (ii) deictic gestures reliably predicted expressive vocabulary size for TD children, but it was baby signs (and not deictic gestures) that predicted expressive vocabulary development for children with DS (Özçaliskan et al., 2016); (iii) non-verbal mental skills predicted language for infants with DS but not for TD children (Mason-Apps et al., 2018); (iv) speech segmentation abilities predicted language outcomes only for TD children (Mason-Apps et al., 2018); and (v) response to joint attention was more predictive of language outcomes in children with DS than in TD peers (Mason-Apps et al., 2018).

This review offers systematic information for researchers, families, and clinicians on language development over time and on language outcomes for individuals with DS. Further research should focus on the yet to be fully studied early predictors of language impairments, and the association between early and later outcomes in DS must be confirmed in larger cohorts. Furthermore, to attain the goal of identifying predictors of language and communication impairments in DS, future studies should combine a set of innovative features, as proposed, for example, within the Predictors of Language Outcomes Project (PLOs)^1^ : (1) inclusion of early measures and later assessments of language abilities for several at-risk groups for language impairments enabling cross-group comparisons; (2) multimethodology approach to a set of potential early predictors of later language outcomes, which combines quantitative and qualitative measures but also other non-invasive methods such as eye gaze, eye tracking, and brain measures; and (3) examination of several language domains at the word and phrase levels (e.g., stress discrimination, word learning, and intonation). This will offer a timely opportunity to promote more effective methods of screening, prevention, early intervention, and diagnosis of language impairments.

In sum, this systematic review shows that there are only a few comprehensive studies that have explored key early predictors of later language acquisition in DS. Although it is difficult to draw strong conclusions based on the relatively limited evidence available, it is becoming increasingly clear that predictors of later language development could be evident in the 5 years of life. Overall, this review confirms that both child-related factors (e.g., maternal education) and prelinguistic communication could predict later language for infants with DS. One important behavioral domain that has received particular attention as a key precursor to language for this clinical population is non-verbal communicative skills such as gestures and signs, together with early vocabulary measures. Furthermore, domain-general processes such as non-verbal cognitive skills have been shown to account for some variations in later language outcomes. However, more studies are needed to identify which factors are the most robust predictors of language development for children with DS, and whether these predictors differ between different clinical populations. A better understanding of the developmental factors that underlie, facilitate, and predict language acquisition in DS would shed light on the nature of this disorder and allow the refinement of targeted early interventions. Such an endeavor would be very relevant for policymakers and service providers to support individuals with DS throughout their lives.

## Author contributions

MF and SF contributed to the conception and design of the work. MF and SC reviewed the abstracts and the manuscript. MF and AV obtained the data from the selected articles. MF prepared the first draft of the manuscript. SF and AV revised the manuscript critically for important intellectual content. MF, AV, and SF revised the last version of the manuscript. All authors contributed to the article and approved the submitted version.

## Footnotes

1
http://labfon.letras.ulisboa.pt/babylab/PLOS/en/
