# Supplementary material for: Early predictors of language outcomes in Down syndrome: A mini-review
Source: Front Psychol. 2022 Sep 14;13:934490. doi: 10.3389/fpsyg.2022.934490 (PMC9515620; doi:10.3389/fpsyg.2022.934490)
Supplement: Supplementary file 1 [file Data_Sheet_1.DOCX]

**Supplementary File: Excluded Papers**

| Study ID | Reference | Reason for exclusion |
| --- | --- | --- |
| 1 | Abed, A. B., Saad, H., Mustpha, R., Chiha, M., & Ben Gamra, S. (2013). Early hearning screening by otoacoustic emissions and auditory brain stem response in Nabeul. *La Tunisie Medicale*, *91*(11), 643–647. http://www.ncbi.nlm.nih.gov/pubmed/24343487 | Does not include measures of language. |
| 2 | Adamson, L. B., Bakeman, R., & Brandon, B. (2015). How parents introduce new words to young children: The influence of development and developmental disorders. *Infant Behavior and Development*, *39*, 148–158. https://doi.org/10.1016/j.infbeh.2015.02.008 | Does not focus on early precursors of language. |
| 3 | Adamson, L. B., Bakeman, R., Deckner, D. F., & Nelson, P. B. (2012). Rating parent–child interactions: Joint engagement, communication dynamics, and shared topics in Autism, Down Syndrome, and typical development. *Journal of Autism and Developmental Disorders*, *42*(12), 2622–2635. https://doi.org/10.1007/s10803-012-1520-1 | Does not focus on early precursors of language. |
| 4 | Arango, P. S., Aparicio, A., & Tenorio, M. (2018). Developmental trajectories of children with Down Syndrome by socio-economic status: the case of Latin America. *Journal of Intellectual Disability Research*, *62*(9), 759–774. https://doi.org/10.1111/jir.12516 | Participants were not followed for a period of 3 months or more. |
| 5 | Aschenbrenner, A. J., Baksh, R. A., Benejam, B., Beresford‐Webb, J. A., Coppus, A., Fortea, J., Handen, B. L., Hartley, S., Head, E., Jaeger, J., Levin, J., Loosli, S. V., Rebillat, A., Sacco, S., Schmitt, F. A., Thurlow, K. E., Zaman, S., Hassenstab, J., & Strydom, A. (2021). Markers of early changes in cognition across cohorts of adults with Down Syndrome at risk of Alzheimer’s disease. *Alzheimer’s & Dementia: Diagnosis, Assessment & Disease Monitoring*, *13*(1). https://doi.org/10.1002/dad2.12184 | Does not focus on early precursors of language. |
| 6 | Audibert, F., & Gagnon, A. (2017). No. 262-Prenatal screening for and diagnosis of aneuploidy in twin pregnancies. *Journal of Obstetrics and Gynaecology Canada*, *39*(9), e347–e361. https://doi.org/10.1016/j.jogc.2017.06.015 | Does not focus on early precursors of language. |
| 7 | Barceló-Coblijn, L., Irurtzun, A., Real Puigdollers, C., López-Navarro, E., & Gomila, A. (2019). How children develop their ability to combine words: A network-based approach. *Adaptive Behavior*, *27*(5), 307–330. https://doi.org/10.1177/1059712319847993 | Does not focus on early precursors of language. |
| 8 | Barker, R. M., Akaba, S., Brady, N. C., & Thiemann-Bourque, K. (2013). Support for AAC use in preschool, and growth in language skills, for young children with developmental disabilities. *Augmentative and Alternative Communication*, *29*(4), 334–346. https://doi.org/10.3109/07434618.2013.848933 | Does not include an early measure (collected before 30 months of age) of language. |

| Study ID | Reference | Reason for exclusion |
| --- | --- | --- |
| 9 | Barstein, J., Martin, G. E., Lee, M., & Losh, M. (2018). A duck wearing boots?! Pragmatic language strategies for repairing communication breakdowns across genetically based neurodevelopmental disabilities. *Journal of Speech, Language, and Hearing Research*, *61*(6), 1440–1454. https://doi.org/10.1044/2018_JSLHR-L-17-0064 | Does not focus on early precursors of language. |
| 10 | Baylis, A. L., & Shriberg, L. D. (2019). Estimates of the prevalence of speech and motor speech disorders in youth with 22q11.2 deletion syndrome. *American Journal of Speech-Language Pathology*, *28*(1), 53–82. https://doi.org/10.1044/2018_AJSLP-18-0037 | Does not focus on early precursors of language. |
| 11 | Baylis, P., & Snowling, M. J. (2012). Evaluation of a phonological reading programme for children with Down Syndrome. *Child Language Teaching and Therapy*, *28*(1), 39–56. https://doi.org/10.1177/0265659011414277 | Does not include an early measure (collected before 30 months of age) of language. |
| 12 | Burgoyne, K., Buckley, S., & Baxter, R. (2021). Speech production accuracy in children with Down syndrome: relationships with hearing, language, and reading ability and change in speech production accuracy over time. *Journal of Intellectual Disability Research*, *65*(12), 1021–1032. https://doi.org/10.1111/jir.12890 | Does not include an early measure (collected before 30 months of age) of language. |
| 13 | Bush, L., Martin, G. E., Landau, E., & Losh, M. (2021). A longitudinal study of parent-child interactions and language outcomes in Fragile X Syndrome and other neurodevelopmental disorders. *Frontiers in Psychiatry*, *12*, 718572. https://doi.org/10.3389/fpsyt.2021.718572 | Does not include an early measure (collected before 30 months of age) of language. |
| 14 | Carr, J. (2012). Six weeks to 45 Years: A longitudinal study of a population with Down Syndrome. *Journal of Applied Research in Intellectual Disabilities*, *25*(5), 414–422. https://doi.org/10.1111/j.1468-3148.2011.00676.x | Does not focus on early precursors of language. |
| 15 | Carr, J., & Collins, S. (2014). Ageing and dementia in a longitudinal study of a cohort with Down Syndrome. *Journal of Applied Research in Intellectual Disabilities*, *27*(6), 555–563. https://doi.org/10.1111/jar.12093 | Does not focus on early precursors of language. |
| 16 | Carr, J., & Collins, S. (2018). 50 years with Down Syndrome: A longitudinal study. *Journal of Applied Research in Intellectual Disabilities*, *31*(5), 743–750. https://doi.org/10.1111/jar.12438 | Does not focus on early precursors of language. |
| 17 | Channell, M. M. (2020). Cross-sectional trajectories of mental state language development in children with Down Syndrome. *American Journal of Speech-Language Pathology*, *29*(2), 760–775. https://doi.org/10.1044/2020_AJSLP-19-00035 | Does not include an early measure (collected before 30 months of age) of language. |
| 18 | Chitayat, D., Langlois, S., & Wilson, R. D. (2017). No. 261-Prenatal screening for fetal aneuploidy in singleton pregnancies. *Journal of Obstetrics and Gynaecology Canada*, *39*(9), e380–e394. https://doi.org/10.1016/j.jogc.2017.06.013 | Does not focus on early precursors of language in infants with DS. |
| 19 | Cleave, P., Bird, E. K.-R., Czutrin, R., & Smith, L. (2012). A longitudinal study of narrative development in children and adolescents with Down Syndrome. *Intellectual and Developmental Disabilities*, *50*(4), 332–342. https://doi.org/10.1352/1934-9556-50.4.332 | Does not include an early measure (collected before 30 months of age) of language. |

| Study ID | Reference | Reason for exclusion |
| --- | --- | --- |
| 20 | Conners, F. A., Tungate, A. S., Abbeduto, L., Merrill, E. C., & Faught, G. G. (2018). Growth and decline in language and phonological memory over two years among adolescents with Down Syndrome. *American Journal on Intellectual and Developmental Disabilities*, *123*(2), 103–118. https://doi.org/10.1352/1944-7558-123.2.103 | Does not include an early measure (collected before 30 months of age) of language. |
| 21 | Coppens-Hofman, MC, van Schrojenstein Lantman-de Valk, HM, & Snik, A. (2012). Speech difficulties and poor speech intelligibility in adults with Down Syndrome: a review of the literature. *Journal of Hearing Science*, *2*(1), 9–16. https://doi.org/10.17430/882708 | The study design is not experimental or observational. |
| 22 | Cuskelly, M., Povey, J., & Jobling, A. (2016). Trajectories of development of receptive vocabulary in individuals with Down Syndrome. *Journal of Policy and Practice in Intellectual Disabilities*, *13*(2), 111–119. https://doi.org/10.1111/jppi.12151 | Does not include an early measure (collected before 30 months of age) of language. |
| 23 | de la Torre, R., de Sola, S., Hernandez, G., Farré, M., Pujol, J., Rodriguez, J., Espadaler, J. M., Langohr, K., Cuenca-Royo, A., Principe, A., Xicota, L., Janel, N., Catuara-Solarz, S., Sanchez-Benavides, G., Bléhaut, H., Dueñas-Espín, I., del Hoyo, L., Benejam, B., Blanco-Hinojo, L., … Dierssen, M. (2016). Safety and efficacy of cognitive training plus epigallocatechin-3-gallate in young adults with Down’s Syndrome (TESDAD): A double-blind, randomised, placebo-controlled, phase 2 trial. *The Lancet Neurology*, *15*(8), 801–810. https://doi.org/10.1016/S1474-4422(16)30034-5 | Does not focus on early precursors of language. |
| 24 | Deckers, S. R. J. M., Van Zaalen, Y., Mens, E. J. M., Van Balkom, H., & Verhoeven, L. (2016). The concurrent and predictive validity of the Dutch version of the Communicative Development Inventory in children with Down Syndrome for the assessment of expressive vocabulary in verbal and signed modalities. *Research in Developmental Disabilities*, *56*, 99–107. https://doi.org/10.1016/j.ridd.2016.05.017 | Does not focus on early precursors of language. |
| 25 | del Hoyo Soriano, L., Thurman, A. J., Harvey, D., Kover, S. T., & Abbeduto, L. (2020). Expressive language development in adolescents with Down Dyndrome and Fragile X Syndrome: change over time and the role of family-related factors. *Journal of Neurodevelopmental Disorders*, *12*(1), 18. https://doi.org/10.1186/s11689-020-09320-7 | Does not include an early measure (collected before 30 months of age) of language. |
| 26 | Draghi, L., & Zampini, L. (2019). The emergence of multiword utterances in children with Down Syndrome. *Clinical Linguistics & Phonetics*, *33*(5), 406–419. https://doi.org/10.1080/02699206.2018.1521871 | Does not include an early measure (collected before 30 months of age) of language. |
| 27 | Fortnum, H., Leighton, P., Smith, M. D., Brown, L., Jones, M., Benton, C., Marder, E., Marshall, A., & Sutton, K. (2014). Assessment of the feasibility and clinical value of further research to evaluate the management options for children with Down Syndrome and otitis media with effusion: a feasibility study. *Health Technology Assessment (Winchester, England)*, *18*(60), 1–147, v–vi. https://doi.org/10.3310/hta18600 | Does not focus on early precursors of language. |

| Study ID | Reference | Reason for exclusion |
| --- | --- | --- |
| 28 | Fuengfoo, A., & Sakulnoom, K. (2014). Clinical abnormalities, early intervention program of Down Syndrome children: Queen Sirikit National Institute of child health experience. *Journal of the Medical Association of Thailand = Chotmaihet Thangphaet*, *97 Suppl 6*, S152-8. http://www.ncbi.nlm.nih.gov/pubmed/25391188 | Does not focus on early precursors of language. |
| 29 | Gilheaney, Ó., Béchet, S., Kerr, P., Kenny, C., Smith, S., Kouider, R., Kidd, R., & Walshe, M. (2018). The prevalence of oral stage dysphagia in adults presenting with temporomandibular disorders: A systematic review and meta-analysis. *Acta Odontologica Scandinavica*, *76*(6), 448–458. https://doi.org/10.1080/00016357.2018.1424936 | The study design is not experimental or observational. |
| 30 | Ginani, C. T. A., Luz, J. R. D. da, Silva, S. V. E., Coppedè, F., & Almeida, M. das G. (2022). Association between MTHFR C677T and A1298C gene polymorphisms and maternal risk for Down Syndrome: A protocol for systematic review and/or meta-analysis. *Medicine*, *101*(3), e28293. https://doi.org/10.1097/MD.0000000000028293 | The study design is not experimental or observational. |
| 31 | Havrilla, J. M., Zhao, M., Liu, C., Weng, C., Helbig, I., Bhoj, E., & Wang, K. (2021). Clinical phenotypic spectrum of 4095 Individuals with Down Syndrome from text mining of electronic health records. *Genes*, *12*(8), 1159. https://doi.org/10.3390/genes12081159 | Does not focus on early precursors of language. |
| 32 | He, Y., Wang, Y., Li, Z., Chen, H., Deng, J., Huang, H., He, X., Zeng, W., Liu, M., Huang, B., & Chen, P. (2020). Clinical performance of non‐invasive prenatal testing for trisomies 21, 18 and 13 in twin pregnancies: A cohort study and a systematic meta‐analysis. *Acta Obstetricia et Gynecologica Scandinavica*, *99*(6), 731–743. https://doi.org/10.1111/aogs.13842 | Does not focus on early precursors of language. |
| 33 | Hulme, C., Goetz, K., Brigstocke, S., Nash, H. M., Lervåg, A., & Snowling, M. J. (2012). The growth of reading skills in children with Down Syndrome. *Developmental Science*, *15*(3), 320–329. https://doi.org/10.1111/j.1467-7687.2011.01129.x | Does not include an early measure (collected before 30 months of age) of language. |
| 34 | Javed, F., Akram, Z., Barillas, A. P., Kellesarian, S. V, Ahmed, H. B., Khan, J., & Almas, K. (2018). Outcome of orthodontic palatal plate therapy for orofacial dysfunction in children with Down Syndrome: A systematic review. *Orthodontics & Craniofacial Research*, *21*(1), 20–26. https://doi.org/10.1111/ocr.12211 | The study design is not experimental or observational. |
| 35 | Jones, D., & Morrison, J. (2016). Preventative therapies and periodontal interventions for Down Syndrome patients. *Evidence-Based Dentistry*, *17*(4), 101–102. https://doi.org/10.1038/sj.ebd.6401198 | Does not focus on early precursors of language. |
| 36 | Joyce, A., & Dimitriou, D. (2017). Sleep-disordered breathing and cognitive functioning in preschool children with and without Down Syndrome. *Journal of Intellectual Disability Research*, *61*(8), 778–791. https://doi.org/10.1111/jir.12387 | Does not include an early measure (collected before 30 months of age) of language. |
| 37 | Kettering, K. R. (2019). “Is down always out?”: The right of Icelandic parents to use preimplantation genetic diagnosis to select for a disability. *The George Washington International Law Review, 51*(4), 1-29. | Does not focus on early precursors of language. |

| Study ID | Reference | Reason for exclusion |
| --- | --- | --- |
| 38 | Lavra-Pinto, B. de, Segabinazi, J. D., & Hübner, L. C. (2014). Consciência fonológica e desenvolvimento da escrita na Síndrome de Down: Um estudo de caso longitudinal. *Revista CEFAC*, *16*(5), 1669–1679. https://doi.org/10.1590/1982-021620147913 | The study design is not experimental or observational. |
| 39 | Laws, G., Brown, H., & Main, E. (2016). Reading comprehension in children with Down Syndrome. *Reading and Writing*, *29*(1), 21–45. https://doi.org/10.1007/s11145-015-9578-8 | Does not include an early measure (collected before 30 months of age) of language. |
| 40 | Lee, M., Bush, L., Martin, G. E., Barstein, J., Maltman, N., Klusek, J., & Losh, M. (2017). A multi-method investigation of pragmatic development in individuals with Down Syndrome. *American Journal on Intellectual and Developmental Disabilities*, *122*(4), 289–309. https://doi.org/10.1352/1944-7558-122.4.289 | Does not include an early measure (collected before 30 months of age) of language. |
| 41 | Linn, K., Sevilla, F., Cifuentes, V., Eugenin, M. I., Río, B., Cerda, J., & Lizama, M. (2019). Development of communicative abilities in infants with Down Syndrome after systematized training in gestural communication. *Revista Chilena de Pediatria*, *90*(2), 175–185. https://doi.org/10.32641/rchped.v90i2.670 | Not in English. |
| 42 | Liogier d’Ardhuy, X., Edgin, J. O., Bouis, C., de Sola, S., Goeldner, C., Kishnani, P., Nöldeke, J., Rice, S., Sacco, S., Squassante, L., Spiridigliozzi, G., Visootsak, J., Heller, J., & Khwaja, O. (2015). Assessment of cognitive scales to examine memory, executive function and language in individuals with Down Syndrome: Implications of a 6-month observational study. *Frontiers in Behavioral Neuroscience*, *9*. https://doi.org/10.3389/fnbeh.2015.00300 | Does not include an early measure (collected before 30 months of age) of language. |
| 43 | Lukowski, A. F., Milojevich, H. M., & Eales, L. (2019). *Cognitive functioning in children with Down Syndrome: Current knowledge and future directions* (pp. 257–289). https://doi.org/10.1016/bs.acdb.2019.01.002 | The study design is not experimental or observational. |
| 44 | Martín-Urda Rodríguez, L., Carchenilla Martín, T., & Moradela Sepúlveda, E. (2019). Eficacia de la intervención lingüística en adolescentes con Síndrome de Down. *Siglo Cero Revista Española Sobre Discapacidad Intelectual*, *50*(1), 43. https://doi.org/10.14201/scero20195014352 | Not in English. |
| 45 | Martin, G. E., Barstein, J., Patel, S., Lee, M., Henry, L., & Losh, M. (2020). Longitudinal analysis of communication repair skills across three neurodevelopmental disabilities. *International Journal of Language & Communication Disorders*, *55*(1), 26–42. https://doi.org/10.1111/1460-6984.12500 | Does not include an early measure (collected before 30 months of age) of language. |
| 46 | Martin, G. E., Losh, M., Estigarribia, B., Sideris, J., & Roberts, J. (2013). Longitudinal profiles of expressive vocabulary, syntax and pragmatic language in boys with Fragile X Syndrome or Down Syndrome. *International Journal of Language & Communication Disorders*, *48*(4), 432–443. https://doi.org/10.1111/1460-6984.12019 | Does not include an early measure (collected before 30 months of age) of language. |
| 47 | Mason-Apps, E., Stojanovik, V., Houston-Price, C., Seager, E., & Buckley, S. (2020). Do infants with Down Syndrome show an early receptive language advantage? *Journal of Speech, Language, and Hearing Research*, *63*(2), 585–598. https://doi.org/10.1044/2019_JSLHR-19-00157 | Statistical analysis does not focus on early precursors of language. |

| Study ID | Reference | Reason for exclusion |
| --- | --- | --- |
| 48 | Monari Martinez, E., & Spada, P. (2022). Multiple choice tests to assess academic achievements in Down Syndrome and Autism Spectrum Disorder. A longitudinal single case study. *International Journal of Disability, Development and Education*, *69*(3), 807–821. https://doi.org/10.1080/1034912X.2020.1740184 | Does not focus on early precursors of language. |
| 49 | Næss, K.-A. B. (2016). Development of phonological awareness in Down Syndrome: A meta-analysis and empirical study. *Developmental Psychology*, *52*(2), 177–190. https://doi.org/10.1037/a0039840 | The study design is not experimental or observational. |
| 50 | Næss, K.-A. B., Lervåg, A., Lyster, S.-A. H., & Hulme, C. (2015). Longitudinal relationships between language and verbal short-term memory skills in children with Down Syndrome. *Journal of Experimental Child Psychology*, *135*, 43–55. https://doi.org/10.1016/j.jecp.2015.02.004 | Does not include an early measure (collected before 30 months of age) of language. |
| 51 | Næss, K.-A. B., Ostad, J., & Nygaard, E. (2021). Differences and similarities in predictors of expressive vocabulary development between children with Down Syndrome and young typically developing children. *Brain Sciences*, *11*(3), 312. https://doi.org/10.3390/brainsci11030312 | Does not include an early measure (collected before 30 months of age) of language. |
| 52 | Nyman, A., Strömbergsson, S., Lindström, K., Lohmander, A., & Miniscalco, C. (2021). Speech and language in 5-year-olds with different neurological disabilities and the association between early and later consonant production: Speech/language in neurological disabilities. *Developmental Neurorehabilitation*, *24*(6), 408–417. https://doi.org/10.1080/17518423.2021.1899327 | Does not include an early measure (collected before 30 months of age) of language. |
| 53 | Peters, I. A., Heetkamp, K. M., Ursem, N. T. C., Steegers, E. A. P., Denktaş, S., & Knapen, M. F. C. M. (2018). Ethnicity and language proficiency differences in the provision of and intention to use prenatal screening for Down’s Syndrome and congenital anomalies. A prospective, non-selected, register-based study in the netherlands. *Maternal and Child Health Journal*, *22*(3), 343–354. https://doi.org/10.1007/s10995-017-2364-2 | Does not focus on early precursors of language. |
| 54 | Pinheiro, D. L. da S. A., Alves, G. Â. D. S., Fausto, F. M. M., Pessoa, L. S. de F., Silva, L. A. da, Pereira, S. M. de F., & Almeida, L. N. A. de. (2018). Effects of electrostimulation associated with masticatory training in individuals with Down Syndrome. *CoDAS*, *30*(3), e20170074. https://doi.org/10.1590/2317-1782/20182017074 | Does not focus on early precursors of language. |
| 55 | Pochon, R., & Declercq, C. (2014). Emotional lexicon understanding and emotion recognition: A longitudinal study in children with Down Syndrome. *Journal of Developmental and Physical Disabilities*, *26*(5), 549–563. https://doi.org/10.1007/s10882-014-9380-6 | Does not focus on early precursors of language. |
| 56 | Pujol, J., Fenoll, R., Ribas-Vidal, N., Martínez-Vilavella, G., Blanco-Hinojo, L., García-Alba, J., Deus, J., Novell, R., & Esteba-Castillo, S. (2018). A longitudinal study of brain anatomy changes preceding dementia in Down syndrome. *NeuroImage: Clinical*, *18*, 160–166. https://doi.org/10.1016/j.nicl.2018.01.024 | Does not focus on early precursors of language. |

| Study ID | Reference | Reason for exclusion |
| --- | --- | --- |
| 57 | Ringenbach, S. D. R., Holzapfel, S. D., Mulvey, G. M., Jimenez, A., Benson, A., & Richter, M. (2016). The effects of assisted cycling therapy (ACT) and voluntary cycling on reaction time and measures of executive function in adolescents with Down Syndrome. *Journal of Intellectual Disability Research*, *60*(11), 1073–1085. https://doi.org/10.1111/jir.12275 | Does not focus on early precursors of language. |
| 58 | Roch, M., & Jarrold, C. (2012). A follow-up study on word and non-word reading skills in Down Syndrome. *Journal of Communication Disorders*, *45*(2), 121–128. https://doi.org/10.1016/j.jcomdis.2011.11.001 | Does not focus on early precursors of language. |
| 59 | Romano, M., Kaiser, A., Lounds-Taylor, J., & Woods, J. (2020). Rates of prelinguistic communication and early symbol use in young children with Down Syndrome: Using a progress-monitoring tool to model growth. *American Journal of Speech-Language Pathology*, *29*(1), 49–62. https://doi.org/10.1044/2019_AJSLP-19-0016 | Statistical analysis does not focus on early precursors of language. |
| 60 | Sacco, S., Bouis, C., Gallard, J., Pichot, A., Blondiaux, E., Marey, I., Dorison, N., Sturtz, F., Cieuta‐Walti, C., Ravel, A., & Mircher, C. (2022). Psychomotor development in infants and young children with Down syndrome—A prospective, repeated measure, post‐hoc analysis. *American Journal of Medical Genetics Part A*, *188*(3), 818–827. https://doi.org/10.1002/ajmg.a.62587 | Does not focus on early precursors of language. |
| 61 | Sagi-Dain, L., Peleg, A., & Sagi, S. (2017). First-trimester crown-rump length and risk of chromosomal aberrations—A systematic review and meta-analysis. *Obstetrical & Gynecological Survey*, *72*(10), 603–609. https://doi.org/10.1097/OGX.0000000000000490 | The study design is not experimental or observational. |
| 62 | Sauvegrain, P., Cognet, M., & Azria, E. (2021). Sociological approach of differential care in Down Syndrome screening based on maternal place of birth. *European Journal of Public Health*, *31*(Supplement_3). https://doi.org/10.1093/eurpub/ckab164.385 | Does not focus on early precursors of language. |
| 63 | Schworer, E., Hoffman, E., & Esbensen, A. (2021). Psychometric evaluation of social cognition and behavior measures in children and adolescents with Down Syndrome. *Brain Sciences*, *11*(7), 836. https://doi.org/10.3390/brainsci11070836 | Does not focus on early precursors of language. |
| 64 | Sepúlveda, E. M., López-Villaseñor, M. L., & Heinze, E. G. (2013). Can individuals with Down Syndrome improve their grammar? *International Journal of Language & Communication Disorders*, *48*(3), 343–349. https://doi.org/10.1111/1460-6984.12002 | Does not focus on early precursors of language. |
| 65 | Smith, M., Manduchi, B., Burke, É., Carroll, R., McCallion, P., & McCarron, M. (2020). Communication difficulties in adults with intellectual disability: Results from a national cross-sectional study. *Research in Developmental Disabilities*, *97*, 103557. https://doi.org/10.1016/j.ridd.2019.103557 | Does not focus on early precursors of language. |
| 66 | Smith, R., Ntsiea, V., Brown, S., & Potterton, J. (2022). Pre-operative neurodevelopmental assessment in young children undergoing cardiac surgery in central South Africa: Feasibility and clinical value. *Cardiovascular Journal of Africa*, *33*, 1–9. https://doi.org/10.5830/CVJA-2021-057 | Does not focus on early precursors of language. |

| Study ID | Reference | Reason for exclusion |
| --- | --- | --- |
| 67 | Spiridigliozzi, G. A., Goeldner, C., Edgin, J., Hart, S. J., Noeldeke, J., Squassante, L., Visootsak, J., Heller, J. H., Khwaja, O., Kishnani, P. S., & Liogier d’Ardhuy, X. (2019). Adaptive behavior in adolescents and adults with Down Syndrome: Results from a 6-month longitudinal study. *American Journal of Medical Genetics Part A*, *179*(1), 85–93. https://doi.org/10.1002/ajmg.a.60685 | Does not focus on early precursors of language. |
| 68 | STARSurg Collaborative and COVIDSurg Collaborative. (2021). Death following pulmonary complications of surgery before and during the SARS-CoV-2 pandemic. *The British Journal of Surgery*, *108*(12), 1448–1464. https://doi.org/10.1093/bjs/znab336 | Does not focus on early precursors of language. |
| 69 | Startin, C. M., Hamburg, S., Hithersay, R., Davies, A., Rodger, E., Aggarwal, N., Al-Janabi, T., & Strydom, A. (2016). The LonDownS adult cognitive assessment to study cognitive abilities and decline in Down Syndrome. *Wellcome Open Research*, *1*, 11. https://doi.org/10.12688/wellcomeopenres.9961.1 | Does not focus on early precursors of language. |
| 70 | Startin, C. M., Lowe, B., Hamburg, S., Hithersay, R., & Strydom, A. (2019). Validating the Cognitive Scale for Down Syndrome (CS-DS) to detect longitudinal cognitive decline in adults with Down Syndrome. *Frontiers in Psychiatry*, *10*. https://doi.org/10.3389/fpsyt.2019.00158 | Does not focus on early precursors of language. |
| 71 | Steele, A., Scerif, G., Cornish, K., & Karmiloff-Smith, A. (2013). Learning to read in williams syndrome and Down Syndrome: Syndrome-specific precursors and developmental trajectories. *Journal of Child Psychology and Psychiatry*, *54*(7), 754–762. https://doi.org/10.1111/jcpp.12070 | Does not include an early measure (collected before 30 months of age) of language. |
| 72 | van Bysterveldt, A., & Gillon, G. (2014). A descriptive study examining phonological awareness and literacy development in children with Down Syndrome. *Folia Phoniatrica et Logopaedica*, *66*(1–2), 48–57. https://doi.org/10.1159/000364864 | Does not include an early measure (collected before 30 months of age) of language. |
| 73 | Watchman, K. (2016). Investigating the lived experience of people with Down Syndrome with Dementia: Overcoming methodological and ethical challenges. *Journal of Policy and Practice in Intellectual Disabilities*, *13*(2), 190–198. https://doi.org/10.1111/jppi.12167 | Does not focus on early precursors of language. |
| 74 | Zis, P., Dickinson, M., Shende, S., Walker, Z., & Strydom, A. (2012). Oxidative stress and memory decline in adults with Down Syndrome: Longitudinal study. *Journal of Alzheimer’s Disease*, *31*(2), 277–283. https://doi.org/10.3233/JAD-2012-120073 | Does not focus on early precursors of language. |
| 75 | Zis, P., McHugh, P., McQuillin, A., Praticò, D., Dickinson, M., Shende, S., Walker, Z., & Strydom, A. (2014). Memory decline in Down Syndrome and its relationship to iPF2alpha, a urinary marker of oxidative stress. *PLoS ONE*, *9*(6), e97709. https://doi.org/10.1371/journal.pone.0097709 | Does not focus on early precursors of language. |
| 76 | Zis, P., Strydom, A., Buckley, D., Adekitan, D., & McHugh, P. C. (2017). Cognitive ability in Down Syndrome and its relationship to urinary neopterin, a marker of activated cellular immunity. *Neuroscience Letters*, *636*, 254–257. https://doi.org/10.1016/j.neulet.2016.11.023 | Does not focus on early precursors of language. |
